# P54 The S* factor: improving outcomes in *Pseudomonas* bacteraemia treatment

**DOI:** 10.1093/jacamr/dlaf230.061

**Published:** 2025-12-04

**Authors:** Miguel Vella

**Affiliations:** Frimley Health NHS Foundation Trust, UK

## Abstract

**Background:**

In 2019, EUCAST redefined its categories of sensitive (S), intermediate (I) and resistant (R). ‘Intermediate’ now indicates likely therapeutic success if higher doses or optimized routes are used. This change particularly affects *Pseudomonas aeruginosa*, where WT isolates are now reported as ‘I’ for most agents. To support clinicians, Berkshire and Surrey Pathology Services (BSPS) introduced the designation S* in laboratory reports while the electronic patient record, EPIC, introduced the term ‘sensitive-increased exposure.’ Although designed to aid clinical decision-making, the change introduces additional terminology that risks confusion at the point of care.

**Objectives:**

(i) To investigate knowledge and understanding of the S*/sensitive-increased exposure category among physicians and pharmacists at Frimley Health NHS Foundation Trust. (ii) To assess whether patients with *Pseudomonas* bacteraemia are receiving appropriately escalated antibiotic dosing in line with EUCAST recommendations.

**Methods:**

We conducted a cross-sectional survey, disseminated by email, to physicians and pharmacists across Wexham Park and Frimley Park hospitals. The questionnaire assessed awareness of the S* category, its interpretation and reported prescribing behaviours. In parallel, we performed a retrospective audit of all adult patients with blood culture–confirmed *Pseudomonas* bacteraemia across both hospitals between January and December 2024. Case notes and prescribing records were reviewed to determine whether ciprofloxacin and/or piperacillin/tazobactam, specifically, were used, and if dosing was standard or appropriately escalated.

**Results:**

A total of 131 responses were received: 100 from physicians and 31 from pharmacists. Among physicians, 69% reported no prior awareness of S*, and 83% were unaware it indicated safe treatment with higher antibiotic doses. Only 29% reported altering their prescribing practice in response to S*. Notably, 33% were more likely to prescribe an antibiotic marked S*, possibly reflecting misinterpretation of S* as a recommendation rather than a caution. Among pharmacists, 48% were unaware of the category, 68% were unsure about dose escalation and 71% did not advise prescribers to modify dosing. The audit identified 70 patients with *Pseudomonas* bacteraemia across both sites in 2024. Of these, 46 (65.7%) were treated with either ciprofloxacin, piperacillin/tazobactam, or both. 30/46 patients (61%) consistently received low doses, 17% were partially escalated and only 24% consistently received correct high doses. Patients at Frimley Park Hospital were significantly more likely to receive appropriate dosing compared to Wexham Park Hospital (*P<*0.001).

**Conclusions:**

Knowledge of the S* category is limited among both doctors and pharmacists, leading to frequent undertreatment of *Pseudomonas* bacteraemia. This gap poses risks for patient outcomes and antimicrobial stewardship. Our findings highlight the need for structured education, clearer microbiology reporting and standardized dosing guidance. In response, we have launched an educational programme across the Trust and plan repeat surveys and audits to evaluate its impact. Improving understanding and practice around the S* category is essential to ensure that EUCAST’s redefinitions translate into improved patient outcomes rather than confusion and underdosing.

